# Transcriptome and Gene Family Analyses Reveal the Physiological and Immune Regulatory Mechanisms of *Channa maculata* Larvae in Response to Nanoplastic-Induced Oxidative Stress

**DOI:** 10.3390/antiox15010125

**Published:** 2026-01-19

**Authors:** Ziwen Yang, Dandan Gao, Yuntao Lu, Yang Zou, Yueying Deng, Luping Liu, Qing Luo, Haiyang Liu, Shuzhan Fei, Kunci Chen, Jian Zhao, Mi Ou

**Affiliations:** 1 School of Fishery, Zhejiang Ocean University, Zhoushan 316022, China; 17707276905@163.com; 2 Key Laboratory of Tropical and Subtropical Fishery Resources Application and Cultivation, Ministry of Agriculture and Rural Affairs, Pearl River Fisheries Research Institute, Chinese Academy of Fishery Sciences, Guangzhou 510380, China; 17633537502@163.com (Y.L.); zy20030430zy@163.com (Y.Z.); dyy12504@163.com (Y.D.); lluping0308@163.com (L.L.); luoqing@prfri.ac.cn (Q.L.); hyliu@prfri.ac.cn (H.L.); feisz@prfri.ac.cn (S.F.); chenkunci@prfri.ac.cn (K.C.); 3 Suqian Institute of Agricultural Sciences, Jiangsu Academy of Agricultural Sciences, Suqian 223802, China; 20240099@jaas.ac.cn; 4 School of Life and Health Sciences, Hunan University of Science and Technology, Xiangtan 411201, China; 5 College of Fisheries and Life Sciences, Shanghai Ocean University, Shanghai 201306, China

**Keywords:** *Channa maculata*, polystyrene nanoplastics, transcriptomics analysis, gene family analysis, histopathology, antioxidant capacity

## Abstract

The increasing accumulation of plastic debris in aquatic environments has raised concerns about the ecotoxicological effects of polystyrene nanoplastics (PSNPs). This study examined PSNPs toxicity during a critical developmental stage by exposing 15 days post-fertilization (dpf) larvae of blotched snakehead (*Channa maculata*), an economically important freshwater fish, to PSNPs concentrations of 0.05–20 mg/L for 15 days. Histopathological analysis showed concentration-dependent damage, including hepatocellular vacuolization (5–10 mg/L) and hepatic sinusoidal dilation (20 mg/L) in the liver, alongside intestinal injuries ranging from villus erosion to rupture (5–20 mg/L). Biochemically, PSNPs triggered a biphasic oxidative response, where superoxide dismutase (SOD) and catalase (CAT) activities peaked at 5 mg/L before declining, while malondialdehyde (MDA) levels exhibited an opposite trend. Transcriptomic analysis and Quantitative real-time PCR (qRT-PCR) indicated that PSNPs disrupted growth, energy metabolism, and immune regulation in *C. maculata* larvae, evidenced by the dysregulation of growth hormone/insulin-like growth factor (GH/IGF) axis genes and up-regulation of immune-related genes. Furthermore, Weighted Gene Co-expression Network Analysis (WGCNA) identified the heterogeneous nuclear ribonucleoproteins (*HNRNP*) gene family as hub genes from the key turquoise module, suggesting that PSNPs interfere with RNA processing and post-transcriptional control. In summary, PSNPs caused multi-level toxicity in *C. maculata* larvae, providing new insights into their ecotoxicological hazards in freshwater ecosystems.

## 1. Introduction

The escalating abundance and spatial proliferation of micro- and nanoplastics (M/NPs) have raised significant concerns regarding their ecological and toxicological hazards to aquatic ecosystems [1]. Current estimates indicate that 1.1 to 4.9 million tons of plastic particles are afloat on the ocean surface [2], a quantity projected to increase without stringent intervention [3]. These contaminants are now ubiquitous in freshwater systems across Europe [4,5,6], North America [7,8], and China [9], with elevated concentrations frequently detected in water bodies experiencing intensive urbanization and industrial pressure. Substantial regional heterogeneity in contamination levels exists. For example, nanoplastic mass concentrations range from 88 to 305 μg/L in Lake Taihu, compared to 29.6–1504.4 μg/L across the Pearl River Basin [10]. This spatial variability is presumably driven by watershed-level factors, including population density, plastic consumption, wastewater treatment efficiency, and hydrodynamic regimes [10]. In freshwater environments, NPs (<1000 nm) originate predominantly from the environmental weathering, mechanical abrasion, and fragmentation of conventional plastic products, leading to a diverse polymer composition primarily comprising polyethylene (PE), polypropylene (PP), polystyrene (PS), polyvinyl chloride (PVC), and polyethylene terephthalate (PET) [11]. Their minute size and pronounced environmental persistence enhance bioavailability, facilitating uptake and bioaccumulation by aquatic organisms, which may ultimately result in systemic, multi-organ toxic effects [12]. For instance, polystyrene nanoplastics (PSNPs) measuring 50 nm can rapidly cross the intestinal barrier of European sea bass (*Dicentrarchus labrax*) and disseminate via the bloodstream to other organs [13]. Similarly, 44 nm PSNPs are continuously taken up by goldfish (*Carassius auratus*), accumulating preferentially in internal tissues, such as the liver and muscle, and ultimately causing genotoxic damage [14]. Furthermore, NPs can infiltrate the brain of zebrafish (*Danio rerio*), inducing size-dependent neurotoxicity, inflammatory responses, and sensory dysfunction [15]. Despite this evidence, toxicological research has predominantly focused on adult fish, with impacts on larval and early life stages remaining less explored, particularly for ecologically sensitive and economically important species. Larval fish are especially vulnerable due to their underdeveloped immune and detoxification systems, often suffering more severe damage from equivalent NP exposures [16]. For example, PSNPs (25 nm) are rapidly absorbed during the early development of *D. rerio*, preferentially accumulating in the yolk sac before translocating to multiple organs and causing developmental defects, oxidative damage, and immunotoxicity [17]. Similarly, microplastics (MPs) exposure induces dose-dependent histopathological lesions, apoptosis, and developmental disruption in Nile tilapia (*Oreochromis niloticus*) larvae [18]. Collectively, these findings underscore the capacity of M/NPs to inflict significant multi-organ toxicity during critical early life stages, threatening the health and stability of freshwater fish populations.

Recent advances in DNA sequencing and omics technologies have revolutionized the study of biological mechanisms. High-throughput RNA sequencing (RNA-seq), in particular, enables comprehensive transcriptomic analysis by identifying differentially expressed genes (DEGs) in response to experimental treatments [19]. This methodology has been widely applied in aquatic toxicology, where transcriptomic analyses have demonstrated that exposure to PSNPs disrupts diverse biological processes in fish, including energy metabolism, immune response, and neurobehavioral function [20,21,22]. These studies provide molecular evidence for PSNPs-induced effects like oxidative stress, nutrient absorption disorders, and metabolic reprogramming, linking them to specific signaling pathways. While RNA-seq effectively characterizes transcriptional responses and identifies enriched pathways, a key limitation is that it often treats gene expression changes as isolated events rather than as components of an integrated regulatory network [23,24]. In reality, these changes occur within coordinated networks. To address this, Weighted Gene Co-expression Network Analysis (WGCNA) is used to identify modules of highly correlated genes and to screen for hub genes central to specific phenotypes [25]. This systems biology approach provides a more integrated perspective, uncovering the co-regulatory architecture and core mechanisms underlying PSNPs-induced stress, and thereby offers a more comprehensive understanding of its complex toxicological mode of action.

Gene family analysis is a pivotal method for deciphering molecular adaptations to environmental stress in fish, such as those induced by M/NPs. Exposure to M/NPs triggers oxidative stress by generating reactive oxygen species (ROS), which activates key signaling pathways like MAPK, Nrf2-ARE, and NF-κB, ultimately leading to immune dysfunction, inflammation, and apoptosis [26]. This mechanistic understanding underscores the value of gene family analysis for uncovering stress responses. Indeed, recent studies link M/NPs exposure to significant expansions, diversification, and differential expression in stress-related gene families, including heat shock proteins (HSPs), Toll-like receptors (TLRs), and antioxidant-related genes [27,28,29]. The teleost-specific whole-genome duplication (TS-WGD) is considered a key genetic basis for this functional diversification, potentially facilitating adaptation to polluted environments [30]. For example, beyond direct chemical stress, multiple homologs of the Heterogeneous Nuclear Ribonucleoprotein (*HNRNP*) gene family in fishes exhibit significant transcriptional and post-transcriptional regulation in response to environmental stressors like salinity change [31]. Integrating toxicological data with the evolutionary patterns of gene families thus provides a deeper understanding of piscine stress responses. This approach not only illuminates adaptive evolution under pollution but also offers a theoretical foundation for identifying molecular markers of stress resistance in aquaculture.

Blotched snakehead (*Channa maculata*), a benthic freshwater fish of high economic value, prized for its rapid growth and high-quality flesh, is a major aquaculture species in China [32]. Recent toxicological research on a related hybrid snakehead (*C. maculata* ♀ × *C. argus* ♂) demonstrated that exposure to PSNPs impairs growth, antioxidant capacity, and immune function [33]. To investigate these effects during a vulnerable life stage, we exposed 15 days post-fertilization (dpf) *C. maculata* larvae to PSNPs in a 15-day waterborne experiment. We then systematically analyzed the physiological, biochemical, and molecular responses, with a focus on stress adaptation and immune regulation. Transcriptomic analysis identified a key gene family among DEGs, and subsequent bioinformatic characterization indicated its central role in mediating PSNPs-induced toxicity. This study provides critical insights into the ecological hazards of PSNPs and a theoretical basis for health management and biomarker development in commercial fish species.

## 2. Materials and Methods

### 2.1. Ethical Approval

All animal procedures were approved by the Animal Ethics Committee of the Pearl River Fisheries Research Institute, Chinese Academy of Fishery Sciences (Approval No. LAEC-PRFRI-2024-08-02) and were conducted in accordance with relevant animal welfare guidelines.

### 2.2. Chemicals and Reagents

Fluorescent PSNPs (spherical, 80 nm diameter, 10 mg/mL) were supplied by BaseLine ChromTech Research Centre (Tianjin, China). The specific characterization data can be referred to in the study by Wang et al. [34]. Commercial assay kits for determining total protein (TP, A045-2-2), superoxide dismutase (SOD, A001-3), catalase (CAT, A007-1), and malondialdehyde (MDA, A003-1) were procured from the Nanjing Jiancheng Bioengineering Institute (Nanjing, China).

### 2.3. Experimental Design and Sampling

A PSNPs exposure experiment was conducted using *C. maculata* larvae obtained from the Fangcun Experiment Station of Pearl River Fisheries Research Institute (Guangzhou, China). Larvae at 15 dpf, which exhibited distinct phototactic and scotophobic behaviors and were capable of preying on *Artemia*, were selected to ensure a developmentally uniform cohort. Healthy individuals were randomly allocated into six experimental groups (300 individuals per group): an unexposed control and five treatment groups exposed to PSNPs at concentrations of 0.05, 0.5, 5, 10, or 20 mg/L. The selection of these concentrations was informed by the findings of Zhang et al. [35] with appropriate adjustments. Each group was maintained in three replicate 30 L glass aquaria, with 100 larvae per aquarium, resulting in a total of 18 experimental units. All larvae were maintained under standardized aquaculture conditions, with water quality parameters maintained as follows: dissolved oxygen at 6~8 mg/L, nitrite < 0.01 mg/L, ammonia nitrogen < 0.5 mg/L, and pH at 7.0~7.5. A fluorescent PSNPs stock suspension (10 mg/mL) was sonicated for 5 min before each dilution to ensure dispersion homogeneity before being added to the aquaria to achieve the target concentrations. The experiment employed a 15-day static-renewal exposure regime, during which half of the test medium was replaced every 24 h. Mortalities were recorded and removed daily. At the end of the exposure period, larvae were fasted for 24 h before sampling. Subsequently, twenty individuals were randomly collected from each replicate aquarium. A subset of these larvae was fixed in 4% paraformaldehyde (Sangon Biotech, Shanghai, China) for subsequent histological analysis, and the remainder were immediately flash-frozen in liquid nitrogen for biochemical and molecular assays.

### 2.4. Histopathological Analysis

To assess PSNPs-induced tissue damage, larval samples were fixed in Bouin’s solution (ABI, Los Angeles, CA, USA) for 24 h at room temperature. Following fixation, tissues were dehydrated through a graded ethanol series (70%, 80%, 90%, and 100%), cleared in xylene, embedded in paraffin, and sectioned at 5 μm thickness. Tissue sections were then stained with hematoxylin and eosin (H&E) and mounted with neutral resin. Histopathological alterations in the liver and intestines were examined and imaged using a light microscope (Nikon, Tokyo, Japan).

### 2.5. Biochemical Analysis

To quantify oxidative stress, pooled samples from each of the three replicates per treatment were homogenized in 0.9% physiological saline to prepare a 10% (*w*/*v*) homogenate. The homogenate was centrifuged at 2500× *g* for 10 min at 4 °C, and the supernatant was collected for oxidative stress biomarker assays. CAT and SOD activities, along with MDA levels, were measured using commercial assay kits according to manufacturers’ protocols. Absorbance for all assays was determined using a SpectraMax i3x microplate reader (Molecular Devices, San Jose, CA, USA).

### 2.6. Transcriptome Sequencing and Differential Expression Analysis

Transcriptomic analysis was conducted to elucidate the molecular mechanisms of the larval stress response to PSNPs. Total RNA was extracted from flash-frozen larval samples using TRIzol reagent (Invitrogen, Waltham, MA, USA). RNA integrity and purity were evaluated using an Agilent 2100 Bioanalyzer (Agilent Technologies, Santa Clara, CA, USA), and concentrations were determined with a NanoDrop ND-2000 spectrophotometer (Thermo Scientific, Waltham, MA, USA). High-quality RNA was used to construct cDNA libraries with the Illumina TruSeq RNA Library Prep Kit (Illumina, San Diego, CA, USA), following the manufacturer’s protocol for cDNA synthesis, end repair, adapter ligation, and PCR amplification. The libraries were sequenced on an Illumina HiSeq 2000 platform at Biomarker Technologies Co., Ltd. (Beijing, China) to generate paired-end reads. Raw reads were processed using FastQC (v0.11.8) (https://www.bioinformatics.babraham.ac.uk/projects/fastqc/, accessed on 20 May 2025)to remove adapter sequences and low-quality bases. The resulting clean reads were aligned to the *C. maculata* reference genome (SRA Accession No. PRJNA730430) [36] with HISAT2 (v2.0.4) (https://daehwankimlab.github.io/hisat2/, accessed on 20 May 2025), and transcript abundance was quantified as the fragments per kilobase of transcript per million mapped reads (FPKM). Differential expression analysis was performed using DESeq2 (v1.30.1) (https://bioconductor.org/packages/release/bioc/html/DESeq2.html, accessed on 20 May 2025), identifying genes with |log_2_ (fold change)| > 1 and false discovery rate (FDR) < 0.001 as DEGs. These DEGs were subsequently subjected to functional enrichment analysis for Gene Ontology (GO) terms and Kyoto Encyclopedia of Genes and Genomes (KEGG) pathways on the BMKCloud platform (http://www.biocloud.net/), and WGCNA (https://cran.r-project.org/package=WGCNA, accessed on 20 May 2025) was performed using the WGCNA package in R v4.5.1 (https://www.r-project.org/).

### 2.7. Quantitative Real-Time PCR (qRT-PCR) Validation

To validate the transcriptome sequencing results, the expression of key genes was analyzed by qRT-PCR. Total RNA was extracted as described previously, and cDNA was synthesized from 1 μg RNA using PrimeScript reverse transcriptase (Takara, Japan, RR037A). Gene expression was analyzed for immune/inflammation-related genes (*IL-8*, *IL-1Β*, *IL-10*, *TOR*, *IκBα*, and *NF-κB*) and growth-related genes (*GHR*, *IGF1-1*, *IGF1-2*, *IGF2*, and *GH*) using SYBR^®^ Green Master Mix (Toyobo, Osaka, Japan) on a CFX96 Real-Time PCR Detection System (Bio-Rad, Hercules, CA, USA). All reactions were run in technical triplicates across two independent biological replicates, with *β-actin* serving as the reference gene. Primer sequences for qRT-PCR are provided in Supplementary Table S1.

### 2.8. Identification and Characterization of the HNRNP Gene Family

The *HNRNP* gene family was identified in the *C. maculata* genome through a dual-method approach. Initial candidates were retrieved via a BLAST (https://blast.ncbi.nlm.nih.gov/Blast.cgi, accessed on 20 May 2025) search (*E*-value < 1 × 10^−5^), with redundancy removed. These candidates were further validated by BLASTN against the NCBI non-redundant (nr) database (https://www.ncbi.nlm.nih.gov/guide/data-software/, accessed on 20 May 2025). A complementary search was performed using the RNP domain (PF00076) from the Pfam database (http://pfam.xfam.org/) with HMMER in TBtools (*E*-value < 0.01), and domain presence was confirmed via Pfam. The resulting unique sequences were retained for characterization. Chromosomal locations were mapped using the Gene Location Visualize tool in TBtools (v1.098) [37], and molecular weight and isoelectric point were predicted via ExPASy Compute pI/Mw (https://web.expasy.org/compute_pi/, accessed on 20 May 2025). Phylogenetic analysis was conducted with MEGA X (v10.2.6) [38] and visualized using iTOL (v6.0) (http://itol.embl.de/, accessed on 20 May 2025). Gene duplication events were assessed using One Step MCScanX [39]. Synteny relationships were analyzed by comparing *C. maculata* with Common carp (*Cyprinus carpio*), Large yellow croaker (*Larimichthys crocea*), Blue tilapia (*Oreochromis aureus*), Rainbow trout (*Oncorhynchus mykiss*), and Atlantic salmon (*Salmo salar*), using the Dual Synteny Plot for MCScanX. Finally, expression profiles of the identified *HNRNP* genes under PSNPs exposure were analyzed in R v4.5.1, and graphical visualization was conducted using the ggplot2 package (v3.4.4) (https://ggplot2.tidyverse.org/).

### 2.9. Data Analysis

Data are presented as mean ± standard deviation (S.D.). For qRT-PCR data, relative gene expression levels were calculated using the 2^−ΔΔCt^ method. Statistical significance among groups was determined by one-way analysis of variance (ANOVA) followed by Duncan’s multiple range test, with *p*-value < 0.05 considered statistically significant.

## 3. Results

### 3.1. Histopathological Analysis

**Figure 1:**
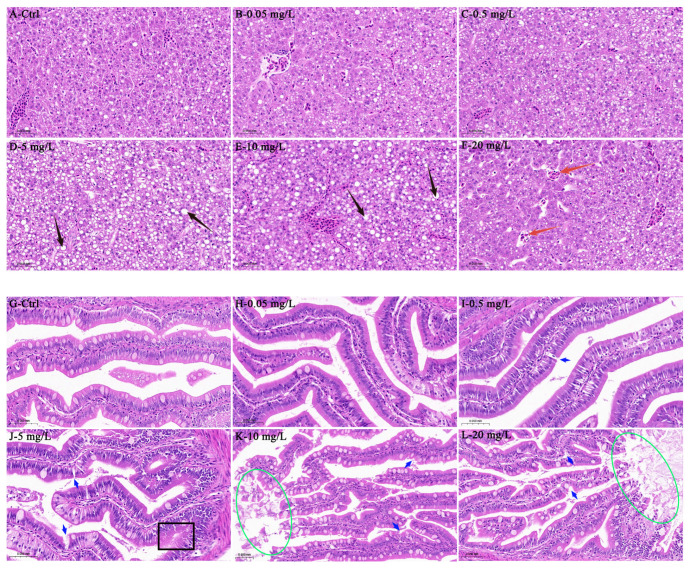
Histopathological alterations in the liver (**A**–**F**) and intestines (**G**–**L**) of *C. maculata* larvae exposed to varying concentrations of PSNPs. Key lesions are marked as follows: the black arrow indicates hepatocellular vacuolization, the red arrow indicates dilation of hepatic sinusoids, the blue arrow indicates intestinal villus rupture, the black square indicates villus erosion, and the green oval indicates villus dissolution.

Histopathological examination of *C. maculata* larvae revealed concentration-dependent tissue damage in the liver and intestines following exposure to PSNPs (Figure 1). In the liver, the control and low-concentration groups (0.05 mg/L and 0.5 mg/L) showed no apparent pathology (Figure 1A–C). Exposure to 5 and 10 mg/L PSNPs induced distinct hepatocellular vacuolization (Figure 1D,E), while the highest concentration of 20 mg/L resulted in slight dilation of the hepatic sinusoids (Figure 1F). Similarly, intestinal morphology was unaffected in the control and 0.05 mg/L exposure group (Figure 1G,H). However, concentrations of 0.5 mg/L and above induced structural injuries that increased in severity with dose (Figure 1I–L). Erosion of the villus wall was evident at 5 mg/L (Figure 1J), progressing to villus rupture and dissolution at 10 and 20 mg/L (Figure 1K,L). Collectively, these results suggest that PSNPs induce significant, concentration-dependent structural damage in both the liver and intestines of *C. maculata* larvae.

### 3.2. Enzyme Activity Analysis

**Figure 2:**
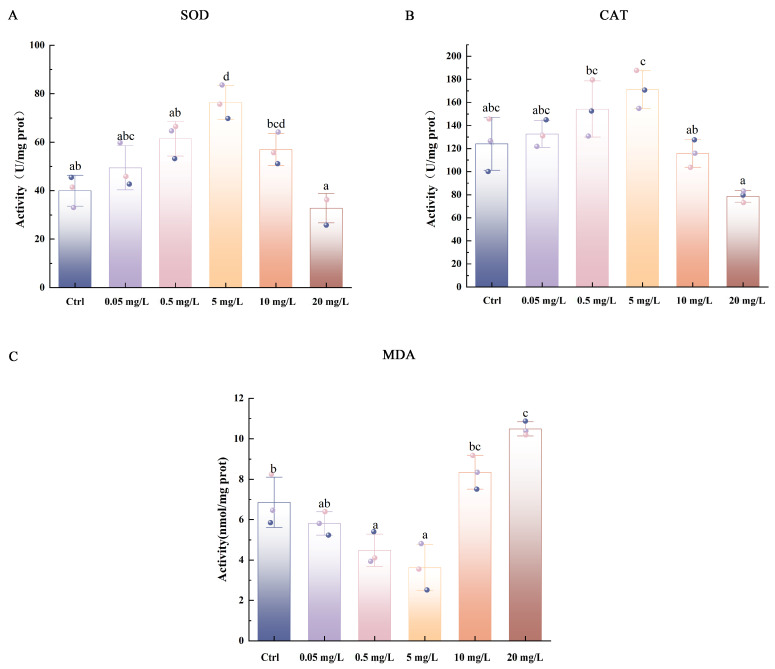
Activities of SOD (**A**), CAT (**B**), and MDA (**C**) in *C. maculata* larvae exposed to different concentrations of PSNPs. Different letters above bars denote significant differences among groups.

The activities of antioxidant enzymes and the level of lipid peroxidation were assessed in 15 dpf *C. maculata* larvae following a 15-day exposure to varying concentrations of PSNPs (Figure 2). SOD activity exhibited a biphasic response, increasing significantly to a peak level approximately two-fold higher than the control at 5 mg/L (*p* < 0.05), before declining to a level marginally below the control at the highest concentration of 20 mg/L (*p* > 0.05) (Figure 2A). CAT activity followed a similar pattern, rising gradually to a maximum at 5 mg/L that was 1.3-fold higher than the control (*p* > 0.05), and then decreasing to a value slightly lower than the control at 20 mg/L (*p* > 0.05) (Figure 2B). In contrast, MDA levels displayed an inverse pattern. A significant decrease was observed at 0.5 and 5 mg/L PSNPs compared to the control (*p* < 0.05), after which levels increased sharply, reaching a peak at 20 mg/L that was approximately 1.5-fold higher than the control (*p* < 0.05) (Figure 2C).

### 3.3. Transcriptome Analysis

Transcriptomic profiling was performed on *C. maculata* larvae from a control group and five groups exposed to PSNPs at concentrations of 0.05, 0.5, 5, 10, and 20 mg/L. After quality control, the 18 RNA-seq samples generated a total of 120.47 Gb of high-quality clean data (Q30 > 96.69%), with an average of 6.01 Gb per sample (SRA Accession No. PRJNA1353071). Clean reads were successfully mapped to the *C. maculata* reference genome, resulting in the identification of 27,528 transcripts, including 24,115 annotated genes and 3413 novel genes. Principal component analysis (PCA) showed clear separation among treatment groups along PC1 (35.19%) and PC2 (17.71%), while biological replicates clustered tightly within each group, indicating high data consistency and reliability. No obvious outliers were detected, supporting the suitability of the dataset for subsequent WGCNA (Figure S1).

Pairwise comparisons between the control and each PSNPs-exposed group identified a total of 33,880 DEGs across all comparisons, including 14,542 up-regulated and 19,338 down-regulated genes (Figure S2). The number of DEGs exhibited an overall non-monotonic increase with increasing PSNPs concentration: 3065 DEGs (1059 up-regulated and 2006 down-regulated) at 0.05 mg/L, 7397 DEGs (3120 up-regulated and 4277 down-regulated) at 0.5 mg/L, 7357 DEGs (3151 up-regulated and 4206 down-regulated) at 5 mg/L, peaking at 8162 DEGs (3997 up-regulated and 4165 down-regulated) at 10 mg/L, and slightly decreasing to 7899 DEGs (3215 up-regulated and 4684 down-regulated) at 20 mg/L.

**Figure 3:**
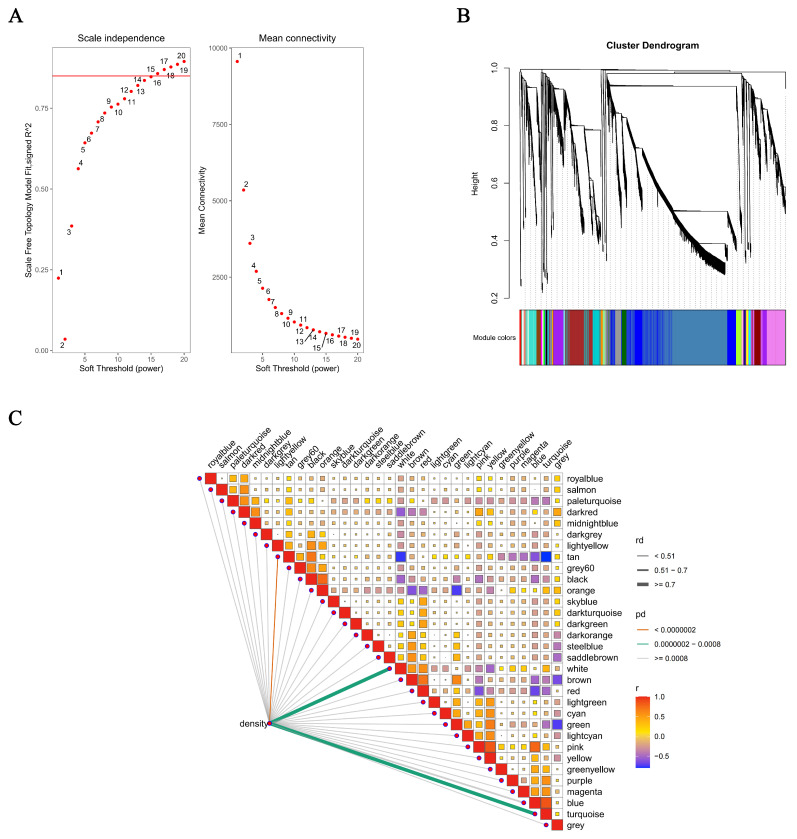
WGCNA of the *C. maculata* larvae transcriptome. (**A**) Analysis of scale-free fit index and mean connectivity for selecting the soft-thresholding power (*β*). (**B**) Cluster dendrogram of genes grouped into co-expression modules using the optimal soft-thresholding power (*β* = 15). (**C**) Module-trait associations showing the correlation between module eigengenes and PSNPs exposure concentrations.

To further explore gene co-expression relationships, WGCNA was conducted on the identified transcripts. Using a soft-thresholding power of *β* = 15 (Figure 3A), genes were clustered into 35 distinct modules based on their expression patterns (Figure 3B). Functional annotation revealed that the tan module was closely associated with muscle growth and development, while the turquoise and white modules were primarily related to metabolism, tissue damage, and neuroendocrine regulation (Figure 3C).

**Figure 4:**
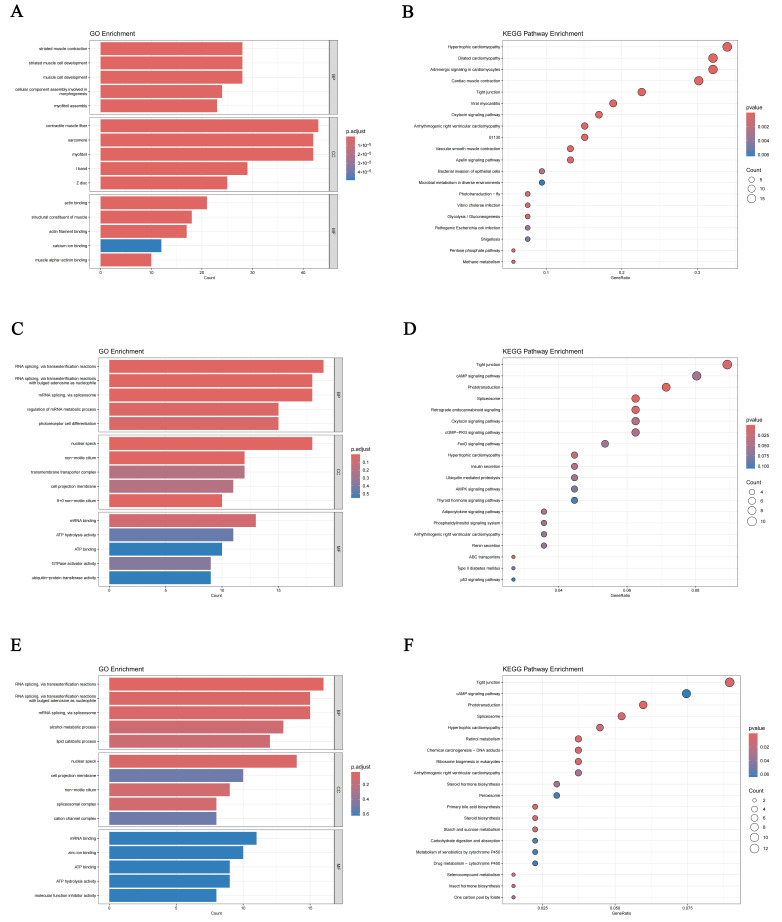
Functional enrichment analyses of key modules in *C. maculata* larvae exposed to PSNPs. (**A**) GO enrichment analysis of the *tan* module. (**B**) KEGG enrichment analysis of the *tan* module. (**C**) GO enrichment analysis of the *turquoise* module. (**D**) KEGG enrichment analysis of the *turquoise* module. (**E**) GO enrichment analysis of the *white* module. (**F**) KEGG enrichment analysis of the *white* module.

**Figure 5:**
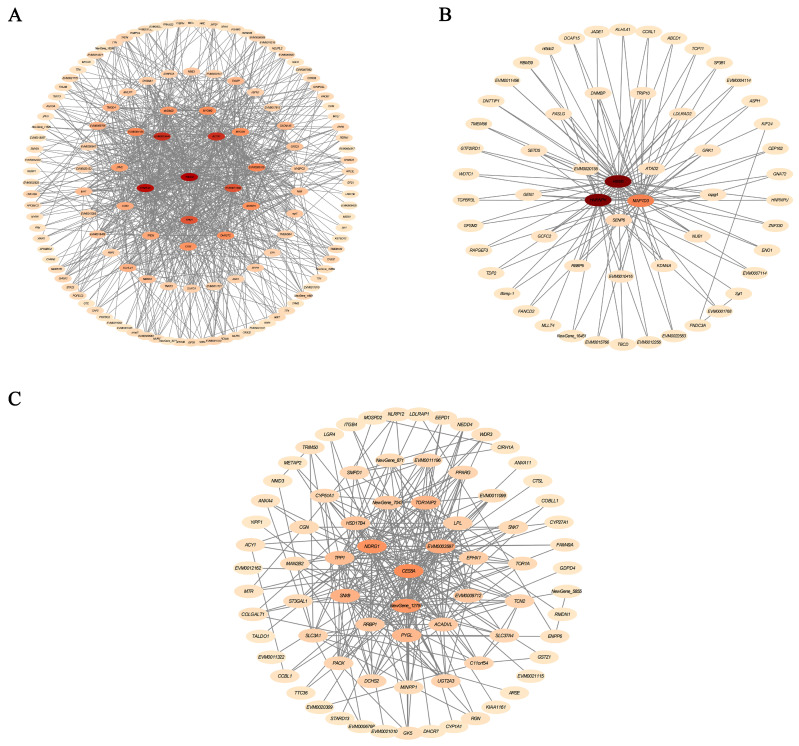
Core gene networks within the *tan* (**A**), *turquoise* (**B**), and *white* (**C**) modules.

In the *tan* module, PSNPs exposure significantly enriched biological processes related to muscle contraction and development (Figure 4A). KEGG pathway analysis further revealed enrichment in cardiac-specific pathways, including hypertrophic cardiomyopathy, dilated cardiomyopathy, adrenergic signaling in cardiomyocytes, and cardiac muscle contraction (Figure 4B). These findings suggest that PSNPs may disrupt cardiac muscle contraction, intercellular junctions, and growth-related signaling. Consistent with this, the hub genes *TNNC2*, *ACTA1*, and *SYNPO2L* were identified, primarily involved in muscle contraction and sarcomere organization (Figure 5A), further indicating that PSNPs exposure impairs muscle development and function. Within the *turquoise* module, PSNPs exposure affected biological processes associated with post-transcriptional regulation and sensory systems. GO enrichment analysis showed significant involvement in RNA splicing and transfer reactions, specifically through the spliceosome, suggesting that PSNPs may influence post-transcriptional gene regulation. Enrichment in photoreceptor cell differentiation indicated potential effects on visual system development (Figure 4C). KEGG analysis supported these findings, showing significant enrichment in tight junctions, cyclic adenosine monophosphate (cAMP) signaling, and phototransduction pathways (Figure 4D), which are critical for cellular barrier functions, signal transduction, and physiological homeostasis. Among these, the hub gene *HNRNPK* was closely related to metabolism and tissue injury (Figure 5B). Notably, the cAMP signaling pathway, a key node in endocrine regulation, may interact with growth pathways such as the growth hormone/insulin-like growth factor (GH/IGF) axis, thereby influencing overall growth and metabolism.

In the *white* module, genes were mainly enriched in RNA splicing, lipid catabolism, and mRNA binding (Figure 4E). KEGG analysis again highlighted tight junctions, phototransduction, and the cAMP signaling pathway (Figure 4F). The hub gene *CES5A* and *NDRG1* were identified within this module. These genes are essential for metabolic regulation, cellular stress responses, protein clearance, and developmental differentiation, which are critical for maintaining neural health and metabolic homeostasis. Furthermore, *NDRG1* has been implicated in monocyte/macrophage differentiation and polarization, and it can suppress NF-κB/STAT3 signaling to downregulate pro-inflammatory cytokines like *IL-6* and *IL-8* (Figure 5C), suggesting a role in immunosuppression and stabilization of inflammatory microenvironment [40].

### 3.4. Gene Expression Analysis

**Figure 6:**
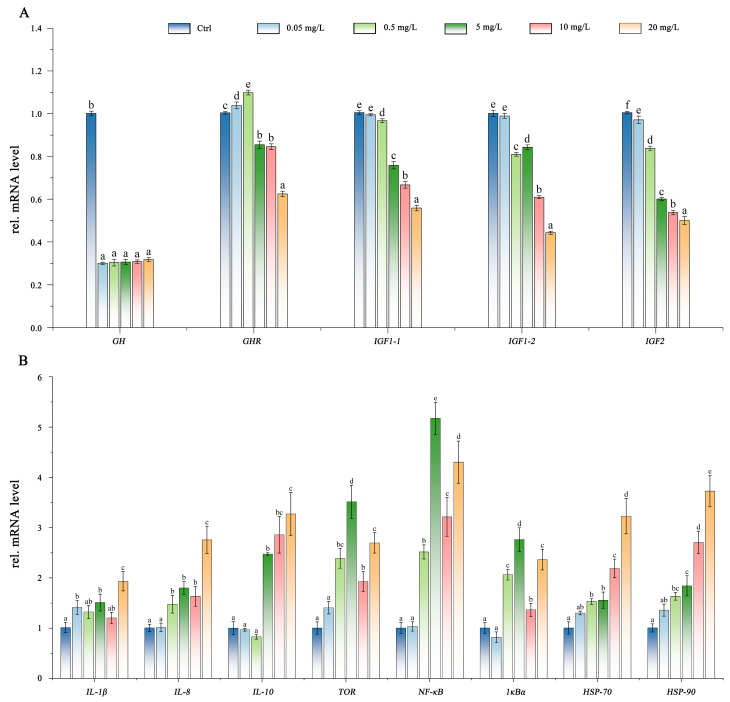
PSNPs exposure modulates the expression of growth-related genes (**A**) as well as immune- and inflammation-related genes (**B**) in *C. maculata* larvae. Expression profiles are shown across a concentration gradient of PSNPs (0, 0.05, 0.5, 5, 10, and 20 mg/L). Letters above bars denote statistically significant differences among treatments (*p* < 0.05).

Expression levels of growth-related genes in *C. maculata* larvae were differentially regulated by PSNP exposure (Figure 6A). Specifically, *GH* expression was significantly suppressed across all exposure groups compared to the control (*p* < 0.05), although this suppression was not dose-dependent (*p* > 0.05). In contrast, *GHR* expression exhibited a biphasic response, characterized by significant up-regulation at low PSNPs concentrations (0.05 and 0.5 mg/L) but significant down-regulation at high concentrations (5, 10, and 20 mg/L). The remaining main genes of the GH/IGF axis (*IGF1-1*, *IGF1-2*, and *IGF2*) showed a consistent and concentration-dependent decrease in expression, with significant suppression observed at 20 mg/L PSNPs group.

To investigate the immunological impact of PSNPs, the expression of key immune- and stress-related genes, including *IL-1β*, *IL-8*, *IL-10*, *TOR*, *NF-κB*, *IκBα*, *HSP-70* and *HSP-90*, was analyzed. Broadly speaking, PSNPs exposure induced a concentration-dependent up-regulation of these genes (Figure 6B). Specifically, compared with the control group, *IL-1β* was significantly elevated at 0.05 mg/L (*p* < 0.05), and a broader set of genes (*IL-8*, *TOR*, *NF-κB*, *IκBα*, *HSP-70*, and *HSP-90*) were significantly up-regulated at 0.5 mg/L (*p* < 0.05). In the high-concentration treatment groups (5, 10, and 20 mg/L), all measured immune-and inflammation-related genes were significantly overexpressed (*p* < 0.05).

### 3.5. Analysis of the HNRNP Gene Family

Transcriptome analysis and WGCNA identified the *turquoise* module as the most strongly correlated with the treatment (*R*^2^ = 0.81). Hub genes within this module were predominantly members of the *HNRNP* gene family, which encode key RNA-binding proteins that regulate essential processes, including pre-mRNA splicing, transport, stability, and translation. These functions are critical for development, growth, and environmental stress adaptation in fish [41]. A total of 23 *HNRNP* genes, representing the complete family in *C. maculata*, were identified and mapped across the genome (Table S2).

#### 3.5.1. Genomic Distribution and Phylogenetic Classification

**Figure 7:**
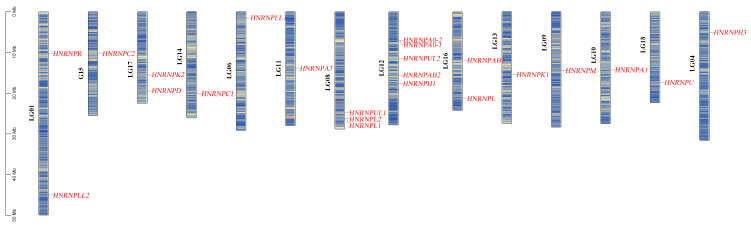
Chromosomal locations of the *HNRNP* gene family in *C. maculata*. Linkage groups (LGs), representing chromosomes, are depicted as vertical blue bars. The 23 identified *HNRNP* genes, mapped to their respective LGs, are shown as red marks.

These 23 *HNRNP* genes were distributed across multiple linkage groups (Figure 7). Specifically, these genes were located on LG01 (*HNRNPLL2*, *HNRNPR*), LG04 (*HNRNPH3*), LG06 (*HNRNPLL1*), LG08 (*HNRNPL1*, *HNRNPUL1*, *HNRNPL2*), LG09 (*HNRNPM*), LG10 (*HNRNPA1*), LG11 (*HNRNPA3*), LG12 (*HNRNPH1*, *HNRNPA0-1*, *HNRNPUL2*, *HNRNPAB2*, *HNRNPA0-2*), LG13 (*HNRNPK1*), LG14 (*HNRNPC1*), LG15 (*HNRNPC2*), LG16 (*HNRNPL1*, *HNRNPAB1*), LG17 (*HNRNPK2*, *HNRNPD*), and LG18 (*HNRNPL3*). This distribution illustrates the widespread genomic localization of this gene family.

Based on phylogenetic reconstruction and subcellular localization profiles, these genes were classified into distinct, well-supported subfamilies (Figure S3), including HNRNPK (HNRNPK1, HNRNPK2), HNRNPU (HNRNPUL1, HNRNPUL2, HNRNPU), HNRNPLL (HNRNPLL1, HNRNPLL2), HNRNPL (HNRNPL1, HNRNPL), HNRNPM, HNRNPH (HNRNPH1, HNRNPH3), HNRNPC (HNRNPC1, HNRNPC2), HNRNPD, HNRNPAB (HNRNPAB1, HNRNPAB2), HNRNPR, and HNRNPA (HNRNPA1, HNRNPA3, HNRNPA0-1, HNRNPA0-2). This clear phylogenetic classification reflects a high degree of evolutionary conservation and suggests subsequent functional diversification among subfamilies.

#### 3.5.2. Evolutionary Conservation Revealed by Synteny Analysis

To elucidate the evolutionary relationships of the HNRNP gene family, a comparative synteny analysis was conducted between *C. maculata* and five other teleost species, including *C. carpio*, *L. crocea*, *O. aureus*, *O. mykiss*, and *S. salar.* The analysis detected a high degree of syntenic conservation, with 10, 13, 13, 13, and 12 syntenic HNRNP gene pairs identified, respectively (Figure S4). The conservation of *HNRNP* genes even after genomic rearrangements across diverse teleost lineages underscores their functional importance in vertebrate evolution.

#### 3.5.3. Expression Profiles in Response to PSNPs Exposure

**Figure 8:**
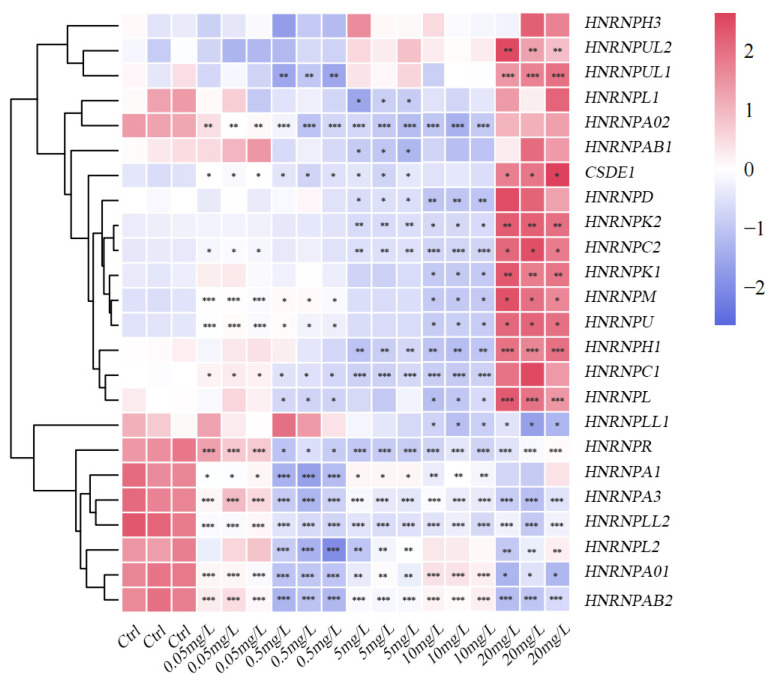
Relative mRNA expression levels of *HNRNP* genes in *C. maculata* larvae following exposure to PSNPs. Asterisks indicate significant differences from the control group (0 mg/L): * indicates statistical significance (*p* < 0.05); ** indicates high statistical significance (*p* < 0.01); and *** indicates extremely significant difference (*p* < 0.001).

The expression profiles of *HNRNP* genes under different concentrations of PSNPs exposure revealed a distinct stress response (Figure 8). Compared with the control group, the expression of nine genes, including *HNRNPUL2*, *HNRNPUL1*, *HNRNPK2*, *HNRNPC2*, *HNRNPK1*, *HNRNPM*, *HNRNPU*, *HNRNPH1*, and *HNRNPL*, was generally suppressed at lower concentrations (0.05–10 mg/L) but was significantly up-regulated at the highest concentration (20 mg/L) (*p* < 0.05). Meanwhile, *HNRNPH3*, *HNRNPAB1*, *HNRNPD*, and *HNRNPC1* displayed an upward trend at 20 mg/L, although the changes were not statistically significant (*p* > 0.05). In contrast, *HNRNPLL1*, *HNRNPR*, *HNRNPA1*, *HNRNPA3*, *HNRNPLL2*, *HNRNPL2*, *HNRNPA01*, and *HNRNPAB2* were more highly expressed in the control group and showed significant down-regulation across all PSNPs exposure levels. These findings suggest that PSNPs-induced environmental stress may suppress the transcriptional activity of a specific subset of *HNRNP* genes, while triggering a potential compensatory or stress-responsive upregulation of another subset at high concentrations, reflecting a complex and differential regulatory response.

## 4. Discussion

The ingestion of PSNPs poses a significant threat to developing aquatic organisms, as their small size facilitates uptake and larval development fosters their systemic translocation [42]. In larva, PSNPs primarily enter into organisms via two routes. First, PSNPs can be ingested through the diet, translocate into the gastrointestinal tract, and be internalized by intestinal epithelial cells through endocytic mechanisms such as pinocytosis and receptor-mediated endocytosis [42,43]. Second, owing to the underdeveloped gill and skin barriers in larva, PSNPs may directly permeate the organism via the gill epithelium or cutaneous mucus, entering the circulatory system [44]. Once internalized, PSNPs can traverse cellular barriers, be widely distributed through systemic circulation [44], and accumulate preferentially in target organs, such as the liver and intestines, ultimately resulting in localized tissue damage [45]. In the liver, PSNPs accumulation induces histopathological alterations such as inflammatory cell infiltration, passive congestion, and sinusoidal dilation [46,47]. Consistent with these findings, *C. maculata* larvae exposed to PSNPs in the present study also exhibited pronounced hepatocellular vacuolation and sinusoidal dilation. These changes may result from PSNPs accumulation interfering with hepatic lipid metabolism and obstructing the microvasculature [48]. Furthermore, the observed concentration-dependent severity of damage is potentially attributable to the ability of PSNPs to inhibit protein synthesis and disrupt energy metabolism [46]. Similarly, the intestines are highly sensitive to PSNPs, with exposure known to elicit inflammation, disrupt the gut microbiota, and lead to systemic metabolic disorders [49]. Our results align with previous research demonstrating intestinal damage in other species, such as villus rupture and enterocyte separation in *D. rerio* [43] and villus abrasion in largemouth bass (*Micropterus salmoides*) [50]. After 15 days of exposure, *C. maculata* larvae exhibited villus damage across all treatment groups (0.5–20 mg/L). The severity of this damage escalated with concentration, progressing from villus wall erosion at 5 mg/L to severe villus rupture and dissolution at 10 and 20 mg/L. In conclusion, the concentration-dependent histological abnormalities observed in both the liver and intestines of exposed fishes serve as early warning signals of impaired health, substantiating that PSNPs accumulation poses a significant toxicological hazard to freshwater fishes by inducing direct tissue damage and potentially disrupting key physiological functions. Beyond organ-specific toxicity, PSNPs exposure may also elicit broader organismal responses. To evaluate these systemic effects, we performed transcriptomic and biochemical analyses on whole-body homogenates. Although this method effectively captures organism-wide regulation, it can dilute responses originating from specific tissues [51]. Consequently, organ-specific pathological damage observed histologically represents direct local toxicity, whereas alterations measured in whole-body samples indicate broader adaptive or pathological responses at the organismal level.

Exposure to MPs has been widely documented to induce oxidative stress responses in aquatic organisms by triggering excessive generation of ROS, as evidenced in multiple fish species, including gilthead seabream (*Sparus aurata*) [52], African catfish (*Clarias gariepinus*) [53], and grass carp (*Ctenopharyngodon idella*) [54]. With decreasing particle size, especially at the nanoscale, biological reactivity increases significantly [42]. This can induce mitochondrial dysfunction, disrupt cellular bioenergetics, and activate stress- and inflammation-related signaling pathways, which promote sustained ROS overproduction [55]. The antioxidant enzymes SOD and CAT work coordinately to mitigate oxidative stress: SOD catalyzes the dismutation of superoxide radicals into hydrogen peroxide (H_2_O_2_), which CAT then decomposes into water and oxygen [56]. However, when ROS production exceeds the scavenging capacity of these antioxidant systems, redox homeostasis is disrupted, leading to the accumulation of oxidative damage in the form of lipid peroxidation, protein oxidation, and DNA damage. Consistent with this, our results indicate that PSNPs provoke a similar oxidative challenge in *C. maculata* larvae, characterized by a biphasic response in the antioxidant enzymes SOD and CAT. Their activities peaked at 5 mg/L, suggesting a compensatory activation of the defense system to maintain cellular homeostasis. However, at higher concentrations (10 and 20 mg/L), a significant decline indicated the suppression of antioxidant capacity. This “low-dose stimulation, high-dose inhibition” pattern aligns with findings in other species. For example, SOD activity in *D*. *rerio* embryos peaks at lower PSNPs concentrations before being inhibited at higher levels [57]. Similarly, CAT activity in *O*. *niloticus* is induced at low or moderate concentrations but suppressed under stronger stress [58]. This suppression likely occurs when the pollutant concentration exceeds a physiological threshold, causing accumulated oxidative damage to impair antioxidant systems through mechanisms like enzyme inactivation. A direct consequence of this overwhelmed defense is lipid peroxidation, which generates toxic products such as MDA that damage cellular membranes [59]. The significant elevation of MDA content in *C. maculata* larvae at high PSNPs concentrations (10 and 20 mg/L) confirms that substantial oxidative damage occurred, a finding consistent with observations in *O. niloticus* and Japanese medaka (*Oryzias latipes*) [60,61]. In summary, PSNPs exposure increases intracellular ROS in *C. maculata* larvae, inducing a concentration-dependent response. Lower concentrations (0.05–5 mg/L) activate the antioxidant defense to preserve redox balance, while higher concentrations (10–20 mg/L) overwhelm these defenses, leading to enzymatic suppression, marked oxidative damage, and a potential collapse of the entire antioxidant system.

Transcriptomic and WGCNA analyses of *C. maculata* larvae exposed to PSNPs identified the disruption of tight junction pathways as a primary effect, indicating a significant impairment of barrier integrity. This finding aligns with established mechanisms of toxicity, as studies on MPs in fishes have similarly shown the disruption of epithelial structures through the modulation of tight junction proteins such as zonula occludens-1 (ZO-1) and Occludin, thereby impairing barrier function [62,63]. Beyond barrier dysfunction, our analysis revealed a significant enrichment of the cAMP and oxytocin signaling pathways, suggesting a broader disruption of neuroendocrine regulation. The enrichment of the cAMP pathway is particularly consequential, as cAMP acts as a central second messenger regulating neurotransmission, metabolic processes, and stress responses, including GHR signaling [64]. This role is corroborated by findings that exogenous pollutants like zinc (Zn) disrupt metabolism in yellow catfish (*Pelteobagrus fulvidraco*) via the cAMP/protein kinase A (PKA) pathway [65]. Meanwhile, the enrichment of the oxytocin signaling pathway implies that PSNPs may interfere with neuroendocrine regulation, which could disrupt behavior, social interaction, and stress modulation in fish. Given the established role of oxytocin in regulating stress, anxiety, and aggression across species [66], this PSNPs-induced neuroendocrine disruption provides a plausible mechanism for the observed energy metabolism disorders and potential neurotoxic effects.

Transcriptomic disturbances in energy metabolism and neuroendocrine signaling likely converge to inhibit the GH/IGF, a central regulator of growth and metabolism in fishes. The suppression of growth, a key endpoint in microplastic toxicity, is frequently attributed to the metabolic burden of detoxification, oxidative stress, and inflammation [52,67]. In addition, microplastics can cause physical damage to the gastrointestinal mucosa, induce “false satiety”, reduce food intake, and impair nutrient absorption, thus disrupting energy homeostasis and leading to growth inhibition [68,69].Consistent with this, the present study found that PSNPs exposure generally suppressed *GH* expression in larvae and induced a dose-dependent decrease in *IGF1-1*, *IGF1-2*, and *IGF2*. The transient up-regulation of *GHR* at low concentrations (0.05 and 0.5 mg/L), which subsequently declined, suggests an early but ultimately overwhelmed compensatory response. These findings indicate that PSNPs indirectly inhibit the GH/IGF axis, leading to growth retardation. Beyond this endocrine disruption, PSNPs also provoked a significant immune and inflammatory response. WGCNA identified key modules enriched with core genes related to immunometabolic processes (e.g., *NDRG1*, *PPARG*, *SNX9*). Correspondingly, gene expression analysis revealed the up-regulation of multiple inflammation- and immunity-related genes, including *IL-1β*, *IL-8*, *IL-10*, *TOR*, *NF-κB*, *IκBα*, *HSP-70*, and *HSP-90* after PSNPs exposure. The increased expression of *HSP-70* and *HSP-90* indicates an activation of the cellular stress defense system, which functions to maintain proteostasis and mediate immune regulation [70,71]. This finding aligns with previous reports that MPs exposure up-regulated *HSP-70* and *HSP-90* expression in Asian sea bass (*Lates calcarifer*) [72]. The up-regulation of the pro-inflammatory cytokines *IL-1β* and *IL-8*, alongside key signaling molecules *NF-κB*, *TOR*, and *IκBα*, points to the activation of inflammatory pathways. Although the NF-κB pathway was not directly enriched in the transcriptomic analysis, the expression changes in its key regulators suggest its indirect involvement in promoting cytokine secretion and mitigating damage [73]. Furthermore, the increased expression of the anti-inflammatory cytokine *IL-10* may provide a negative feedback mechanism to curb excessive inflammation and maintain immune homeostasis. Taken together, *C. maculata* larvae exposed to PSNPs may mount a coordinated immune response, dynamically balancing pro- and anti-inflammatory signals to preserve physiological stability.

To further validate our transcriptomic results, we focused on the *HNRNP* gene family, specifically the significantly enriched *HNRNPK* from the key *turquoise* module. This family of RNA-binding proteins mediates post-transcriptional responses to environmental stress [74]. Consistent with this role, *HNRNP* members facilitate adaptive responses in fishes, such as osmotic adaptation in *O. mykiss* [31] and cold stress response in *O. niloticus* [75]. In our study, the significant up-regulation of *HNRNPL* at the highest PSNPs concentration (20 mg/L) suggests its involvement in similar stress-adaptive mechanism. Furthermore, given that *HNRNPL* impairs muscle cell development and myotube formation under pathological conditions [76], its PSNPs-induced up-regulation in *C. maculata* larvae may contribute to impaired muscle development and growth in larval fish, a mechanism warranting further investigation. Beyond their developmental roles, *HNRNP* proteins play a critical and dual role in host–virus interactions. While they are essential for maintaining cellular homeostasis, they are frequently co-opted by viruses to facilitate replication [77]. Our data indicate that PSNPs exposure significantly dysregulates the expression of several *HNRNP* members implicated in viral pathogenesis, suggesting a potential mechanism for compromised host immunity. For instance, *HNRNPA/B*, which regulates genome replication and translation for viruses like spring viremia of carp virus (SVCV) and cyprinid herpesvirus 3 (CyHV3) [78], was significantly altered under high-concentration PSNPs exposure. This dysregulation could consequently impair intrinsic antiviral defenses. The interplay between *HNRNP* proteins and viral infection is notably complex. During snakehead vesiculovirus (SHVV) infection, viral leader RNA binds to host proteins *HNRNPA3* and *CSDE1*, and its overexpression promotes viral replication [79]. In contrast, our study found that PSNPs exposure significantly down-regulated *HNRNPA3* expression while markedly up-regulating *CSDE1*. This opposing pattern of regulation may reflect a compensatory host response under stress conditions, suggesting its involvement in antiviral defense or inflammatory processes. Therefore, microplastic-induced stress may interfere with the cooperative regulation of these proteins, thereby disrupting host–virus interactions and immune homeostasis. The role of *HNRNPA1* provides additional insight into how PSNPs might increase viral susceptibility. *HNRNPA1* is known to negatively regulate SHVV replication, as its knockdown promotes viral replication while its overexpression inhibits viral amplification [80]. Our finding that *HNRNPA1* expression was significantly lower in the highest PSNPs exposure group (20 mg/L) compared to the control implies that such exposure could weaken the intrinsic antiviral capacity of larval fish, rendering them more vulnerable to infection. Overall, the *HNRNP* gene family exhibits a significant transcriptional response to PSNPs stress. These changes are implicated in the observed developmental abnormalities and inflammatory responses, and they are likely to affect host immune function and disease resistance. Our findings position *HNRNPs* as key regulatory factors in the host’s response to environmental stress, highlighting their potential as biomarkers in environmental toxicology or as targets for therapeutic intervention.

## 5. Conclusions

This study systematically investigated the physiological, biochemical, molecular, and histological effects of PSNPs on *C. maculata* larvae. The results demonstrated that PSNPs exposure significantly disrupted fish growth and development, energy metabolism, immune regulation, and tissue structural stability through multiple mechanisms. The differential expressions of the *HNRNP* gene family under varying exposure concentrations suggested that PSNPs toxicity may operate at a molecular-level by disrupting RNA processing and transcriptional regulation. Collectively, these findings indicate that PSNPs present a significant physiological and molecular toxicological hazard to aquatic organisms. Future research should integrate multi-omics analyses with long-term exposure experiments to elucidate the comprehensive ecological impacts of microplastic pollution from a systems biology perspective, thereby providing scientific foundation for environmental hazard assessment and management strategies.

## Data Availability

The original data presented in this study are publicly available in the NCBI database at https://www.ncbi.nlm.nih.gov/guide/data-software/, with accession No. PRJNA1353071, accessed on 27 October 2025.

## Supplementary Materials

The following supporting information can be downloaded at: https://www.mdpi.com/article/10.3390/antiox15010125/s1. Figure S1. Principal component analysis (PCA) of transcriptomic profiles from *C. maculata* larvae exposed to PSNPs. Figure S2. DEGs between the control and PSNPs-exposed groups. Figure S3. Phylogenetic analysis and classification of HNRNP proteins. The tree was constructed using the Neighbor-Joining method in MEGA X with 1000 bootstrap replicates. Bootstrap values are indicated by circles at branch nodes. HNRNP proteins from *C. maculata* are highlighted with red stars. Distinct clades, corresponding to established HNRNP subfamilies, are color-coded. Figure S4. Evolutionary conservation of *HNRNP* genes revealed by comparative synteny. Circos plots display syntenic relationships with *C. carpio* (A), *L. crocea* (B), *O. aureus* (C), *O. mykiss* (D), and *S. salar* (E). Gray lines in the background represent overall syntenic blocks between genomes, while red lines specifically connect syntenic *HNRNP* gene pairs. Table S1. The primers involved in qRT-PCR. Table S2. Sequence information of HNRNP gene family in *C. maculata*, sourced from a genome-wide identification analysis of its response to PSNPs.
