# Supplementary material for: Transcriptome and Gene Family Analyses Reveal the Physiological and Immune Regulatory Mechanisms of Channa maculata Larvae in Response to Nanoplastic-Induced Oxidative Stress
Source: Antioxidants (Basel). 2026 Jan 19;15(1):125. doi: 10.3390/antiox15010125 (PMC12838143; doi:10.3390/antiox15010125)
Supplement: Supplementary file 1 [file antioxidants-15-00125-s001.zip › antioxidants-4041249-supplementary.pdf]

Figure S1. Principal component analysis (PCA) of transcriptomic profiles from *C. maculata* larvae exposed to PSNPs.

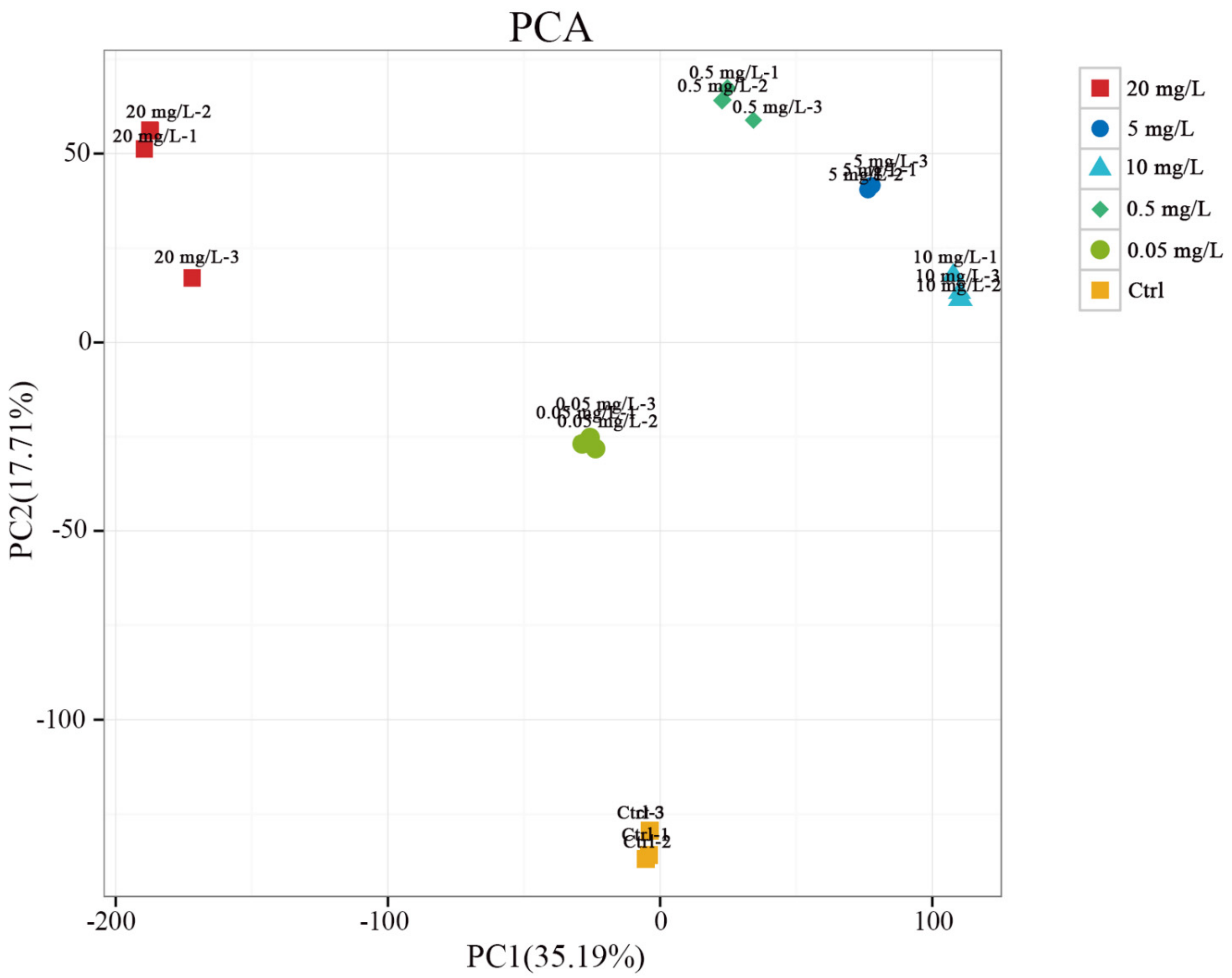

Figure S2. DEGs between the control and PSNPs-exposed groups.

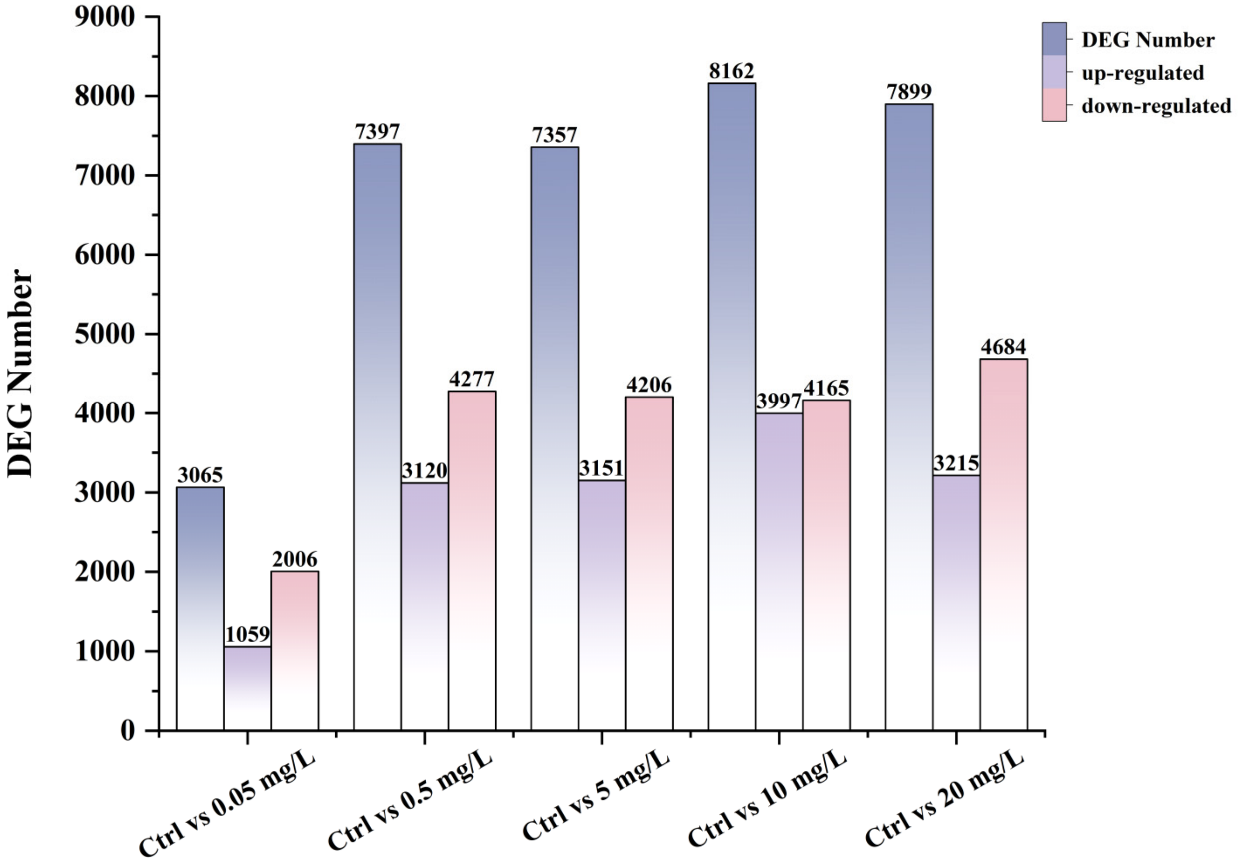

Figure S3. Phylogenetic analysis and classification of HNRNP proteins. The tree was constructed using the Neighbor-Joining method in MEGA X with 1,000 bootstrap replicates. Bootstrap values are indicated by circles at branch nodes. HNRNP proteins from C. maculata are highlighted with red stars. Distinct clades, corresponding to established HNRNP subfamilies, are color-coded.

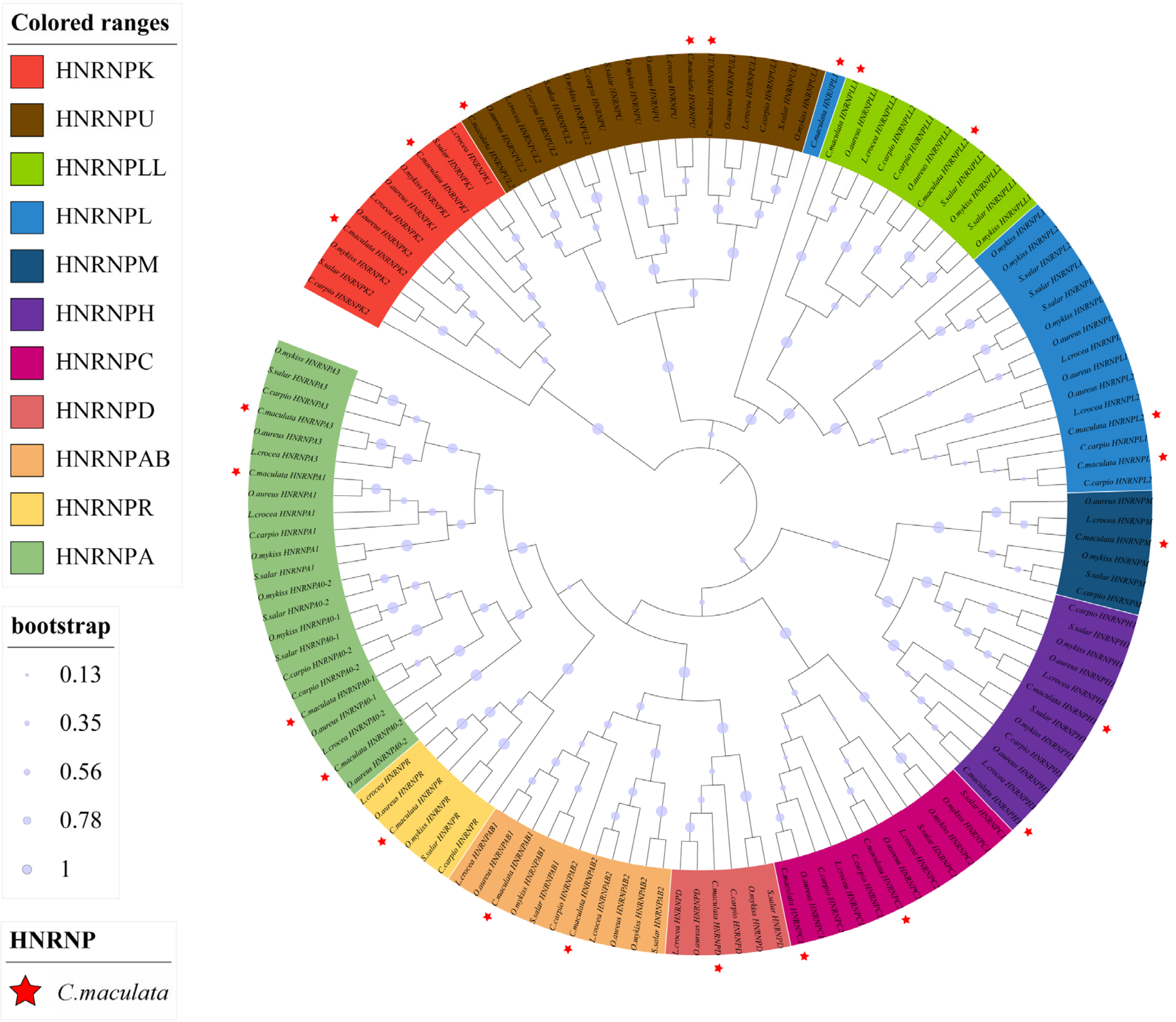

Figure S4. Evolutionary conservation of HNRNP genes revealed by comparative synteny. Circos plots display syntenic relationships with C. carpio (A), L. crocea (B), O. aureus (C), O. mykiss (D), and S. salar (E). Gray lines in the background represent overall syntenic blocks between genomes, while red lines specifically connect syntenic HNRNP gene pairs.

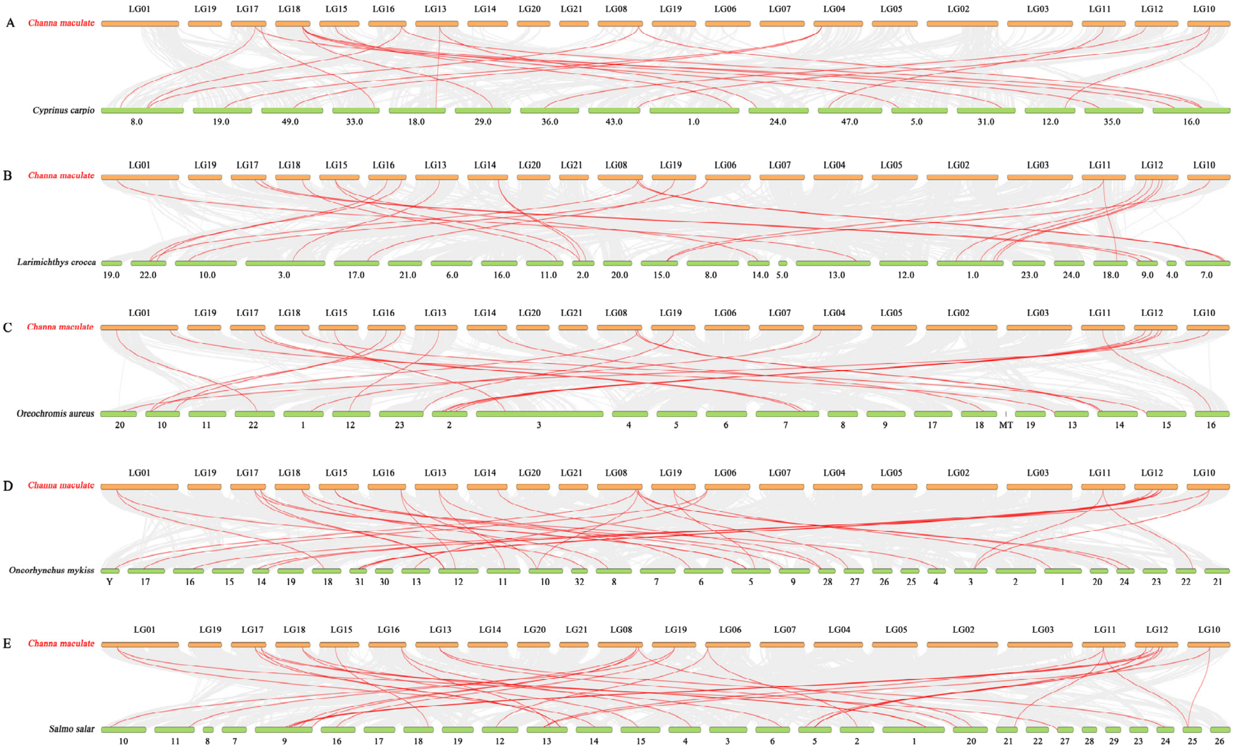

Table S1. The primers involved in qRT-PCR.

Type	Gene name	Primer name	Sequences (5'-3')
Reference gene	*β-actin*	β-actin-F	GCAAGCAGGAGTATGATGAG
		β-actin-R	TTGGGATTGTTTCAGTCAGT
Growth-related gene	*GHR*	GHR-DL-F	ACAGGAACCACACAGCCATT
		GHR-DL-R	GCATCCAGCCCATTTCAAC
	IGF1-1	IGF1-1-DL-F	CGCTCTTTCCTCTCAGTGGC
		IGF1-1-DL-R	CAATGGCTGAGCCCAATGAC
	IGF1-2	IGF1-2-DL-F	GTTTGTGTGTGGAGACAGAGGC
		IGF1-2-DL-R	GCACGCACAGAGTGAGTTGG
	IGF2	IGF2-DL-F	GTCTTCGTCCAGTCGTTCGC
		IGF2-DL-R	TGTTGCCCCTGCTGGTTG
	GH	GH-DL-F	CCGATTACATTATCAGCCCTATTG
		GH-DL-R	AGTAGTTTTCATACGGAGCGAGC
Inflammation-related gene	*IL-1β*	IL-1β-DL-F	GACACGATGCGATTCCTATTCT
		IL-1β-DL-R	CACTGGGCAGTCTTCTCGGA
	IL-8	IL-8-DL-F	GAGTCTGAGCAGCCTGGGAGT
		IL-8-DL-R	CTGTTCGCCGGTTTTCAGTG
	IL-10	IL-10-DL-F	TGGCAGTGAAGAAGACAT
		IL-10-DL-R	CTTTGAAGTGCTCAGGGA
	TOR	TOR-DL-F	GAGCCTCTCTCATCCTCACCAC
		TOR-DL-R	GATTCATTCCTTTCTCTTTAGCCA
	NF-κB	NF-kB-DL-F	CAGCCAAAACCAAGAGGGAT
		NF-kB-DL-R	TCGGCTTCGTAGTAGCCATG
	IκBα	IκBα-DL-F	AAAATGTTACCGTGCCAGGAC
		IκBα-DL-R	ATGTATCACCGTCGTCAGTC
	HSP-70	HSP-70-F	ATTTTGAATGTGTCTGCGGT
		HSP-70-R	ACTTGCTGATGATGGGGTTA
	HSP-90	HSP-90-F	TGTATGTCAGGAGGGTGTTT
		HSP-90-R	TAGATTGATTTCTGGTTTTC

Table S2. Sequence information of *HNRNP* gene family in *C. maculata*, sourced from a genome-wide identification analysis of its response to PSNPs.

Gene_name	Chromosome location	CDS length (bp)	Exon number	Protein molecular weight (Da)	Isoelectric point	ID in *C. maculata* genome
HNRNPLL2	LG01	1893	11	51908.61	8.47	EVM0006823.1
HNRNPR	LG01	1035	11	70598.86	8.4	EVM0020103.1
HNRNPH3	LG04	945	9	44078.26	8.92	EVM0015295.1
HNRNPLL1	LG06	1296	16	59511.27	8.28	EVM0006014.1
HNRNPL1	LG08	1407	12	36458.17	7.06	EVM0000095.1
HNRNPUL1	LG08	954	9	99620.61	4.77	EVM0019158.1
HNRNPL2	LG08	1626	13	52126.66	5.86	EVM0022315.1
HNRNPM	LG09	1155	11	75606.37	9.13	EVM0009364.2
HNRNPA1	LG10	1005	9	35772.94	9.01	EVM0005876.1
HNRNPA3	LG11	2628	15	41033.9	8.73	EVM0000911.1
HNRNPH1	LG12	1416	13	44128.99	6.61	EVM0000654.1
HNRNPA0-1	LG12	993	7	31790.01	8.64	EVM0005228.1
HNRNPUL2	LG12	1224	10	71919.64	7.28	EVM0005906.1
HNRNPAB2	LG12	993	7	36602.78	5.35	EVM0019237.1
HNRNPA0-2	LG12	1920	11	31469.56	8.51	EVM0021901.1
HNRNPK1	LG13	900	1	48386.88	6.65	EVM0001489.1
HNRNPC1	LG14	951	9	35542.63	4.8	EVM0010091.2
HNRNPC2	LG15	1590	14	37965.91	5.34	EVM0013947.2
HNRNPL	LG16	1314	17	58315.27	6.17	EVM0010899.1
HNRNPAB1	LG16	2115	16	35143.45	5.29	EVM0012553.1
HNRNPK2	LG17	1005	10	47630.45	8.21	EVM0002368.2
HNRNPD	LG17	2415	14	35305.93	5.41	EVM0021399.1
HNRNPU	LG18	1212	10	88972.66	5.31	EVM0015275.1
